# *N*-Benzyl-*N*-ethyl­morpholinium chloride

**DOI:** 10.1107/S1600536808041846

**Published:** 2008-12-13

**Authors:** Yan-Jiang Bian

**Affiliations:** a Faculty of Chemistry and Material Science, Langfang Teachers’ College, Hebei, Langfang 065000, People’s Republic of China

## Abstract

In the crystal structure of the title compound, C_13_H_20_NO^+^·Cl^−^, the morpholine ring is in a chair conformation and the mol­ecules are linked by weak inter­molecular C—H⋯Cl hydrogen bonding.

## Related literature

For details of the importance of quaternary morpholine halides see: Kim *et al.* (2005 ▶, 2006 ▶).
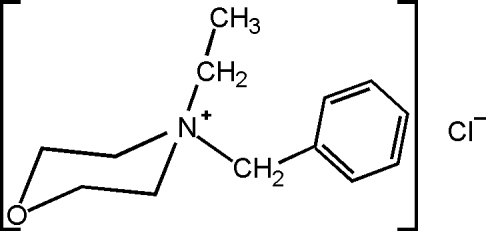


## Experimental

### 

#### Crystal data

C_13_H_20_NO^+^·Cl^−^
*M* *_r_* = 241.75
Monoclinic, 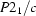

*a* = 13.179 (3) Å
*b* = 8.4176 (17) Å
*c* = 12.255 (3) Å
β = 108.48 (3)°
*V* = 1289.5 (4) Å^3^
*Z* = 4
Mo *K*α radiation
μ = 0.28 mm^−1^
*T* = 113 (2) K
0.16 × 0.16 × 0.06 mm

#### Data collection

Rigaku Saturn CCD area-detector diffractometer
Absorption correction: multi-scan (*CrystalClear*; Rigaku/MSC, 2005 ▶) *T*_min_ = 0.957, *T*_max_ = 0.984
7151 measured reflections
2266 independent reflections
2017 reflections with *I* > 2σ(*I*)
*R*_int_ = 0.031

#### Refinement

*R*[*F* ^2^ > 2σ(*F* ^2^)] = 0.032
*wR*(*F* ^2^) = 0.090
*S* = 1.07
2266 reflections
146 parameters
H-atom parameters constrained
Δρ_max_ = 0.21 e Å^−3^
Δρ_min_ = −0.19 e Å^−3^

### 

Data collection: *CrystalClear* (Rigaku/MSC, 2005 ▶); cell refinement: *CrystalClear*; data reduction: *CrystalClear*; program(s) used to solve structure: *SHELXS97* (Sheldrick, 2008 ▶); program(s) used to refine structure: *SHELXS97* (Sheldrick, 2008 ▶); molecular graphics: *SHELXTL* (Sheldrick, 2008 ▶); software used to prepare material for publication: *SHELXTL*

## Supplementary Material

Crystal structure: contains datablocks I, global. DOI: 10.1107/S1600536808041846/nc2129sup1.cif

Structure factors: contains datablocks I. DOI: 10.1107/S1600536808041846/nc2129Isup2.hkl

Additional supplementary materials:  crystallographic information; 3D view; checkCIF report

## Figures and Tables

**Table 1:** Hydrogen-bond geometry (Å, °)

*D*—H⋯*A*	*D*—H	H⋯*A*	*D*⋯*A*	*D*—H⋯*A*
C1—H1*B*⋯Cl1^i^	0.99	2.74	3.4724 (16)	131
C5—H5*B*⋯Cl1^ii^	0.99	2.61	3.5500 (16)	158
C11—H11⋯Cl1	0.95	2.66	3.5501 (16)	156
C12—H12*B*⋯Cl1^iii^	0.99	2.61	3.5062 (16)	151

Symmetry codes: (i) 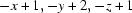; (ii) 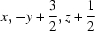; (iii) 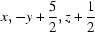.

**Fig. 1.**
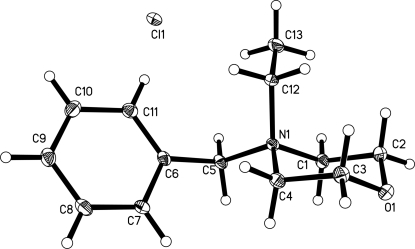
Crystal structure of the title compound with the atom-numbering scheme and displacement ellipsoids drawn at the 30% probability level.

## supplementary crystallographic information

### Comment

Quaternary morpholine halides are valuable precursors for the preparation of ionic liquids by ion metathesis (Kim *et al.*,2005). Ionic liquids based on the morpholinium cation are favored becaused of their low cost, easy synthesis, and electrochemical stability (Kim *et al.*,2006). Here we report a new structure of this class of compounds.

In the crystal structure the morpholine ring adopts a chair conformation (Fig. 1). The cations and anions are connected via weak C—H···Cl hydrogen bonding into a three-dimensional network (Tab 1).

### Experimental

Benzyl chloride(0.12 mol) was added to a solution of 4-ethylmorpholine(0.1 mol) in 20 ml of acetonitrile under stirring. The mixture was stirred at 60 °C for 5 h. The solvent was removed under reduced pressure. The remaining brownish, viscous liquid crystallized slowly at room temperature in a mixture of ethanol and acetone [1/20(*v*/*v*)]. Single-crystals were obtained by slow evaporation of the solvewnt from a solution in a mixture of ethanol and acetone [1/20(*v*/*v*)].

### Refinement

The H atoms were positioned with idealized geometry and were refinement isotropic using a riding model with C–H = 0.96–0.97 Å and *U*_iso_ (H) = 1.2 *U*_eq_ (C) for aromatic and methylene H atoms as well as *U*_iso_(H) = 1.5*U*_eq_(C) for methyl H atoms.

### Crystal data

**Table:**

C_13_H_20_NO^+^·Cl^−^	*F*(000) = 520
*M**_r_* = 241.75	*D*_x_ = 1.245 Mg m^−^^3^
Monoclinic, *P*2_1_/*c*	Mo *K*α radiation, λ = 0.71073 Å
*a* = 13.179 (3) Å	Cell parameters from 3736 reflections
*b* = 8.4176 (17) Å	θ = 1.6–27.9°
*c* = 12.255 (3) Å	µ = 0.28 mm^−^^1^
β = 108.48 (3)°	*T* = 113 K
*V* = 1289.5 (4) Å^3^	Prism, colorless
*Z* = 4	0.16 × 0.16 × 0.06 mm

### Data collection

**Table:**

Rigaku Saturn CCD area-detector diffractometer	2266 independent reflections
Radiation source: rotating anode	2017 reflections with *I* > 2σ(*I*)
confocal	*R*_int_ = 0.031
ω and φ scans	θ_max_ = 25.0°, θ_min_ = 2.9°
Absorption correction: multi-scan (*CrystalClear*; Rigaku/MSC, 2005)	*h* = −15→14
*T*_min_ = 0.957, *T*_max_ = 0.984	*k* = −9→10
7151 measured reflections	*l* = −7→14

### Refinement

**Table:**

Refinement on *F*^2^	Primary atom site location: structure-invariant direct methods
Least-squares matrix: full	Secondary atom site location: difference Fourier map
*R*[*F*^2^ > 2σ(*F*^2^)] = 0.032	Hydrogen site location: inferred from neighbouring sites
*wR*(*F*^2^) = 0.090	H-atom parameters constrained
*S* = 1.07	*w* = 1/[σ^2^(*F*_o_^2^) + (0.0545*P*)^2^ + 0.1492*P*] where *P* = (*F*_o_^2^ + 2*F*_c_^2^)/3
2266 reflections	(Δ/σ)_max_ = 0.002
146 parameters	Δρ_max_ = 0.21 e Å^−^^3^
0 restraints	Δρ_min_ = −0.19 e Å^−^^3^

### Special details

**Table:**

Geometry. All e.s.d.'s (except the e.s.d. in the dihedral angle between two l.s. planes) are estimated using the full covariance matrix. The cell e.s.d.'s are taken into account individually in the estimation of e.s.d.'s in distances, angles and torsion angles; correlations between e.s.d.'s in cell parameters are only used when they are defined by crystal symmetry. An approximate (isotropic) treatment of cell e.s.d.'s is used for estimating e.s.d.'s involving l.s. planes.
Refinement. Refinement of *F*^2^ against ALL reflections. The weighted *R*-factor *wR* and goodness of fit *S* are based on *F*^2^, conventional *R*-factors *R* are based on *F*, with *F* set to zero for negative *F*^2^. The threshold expression of *F*^2^ > σ(*F*^2^) is used only for calculating *R*-factors(gt) *etc*. and is not relevant to the choice of reflections for refinement. *R*-factors based on *F*^2^ are statistically about twice as large as those based on *F*, and *R*- factors based on ALL data will be even larger.

### Fractional atomic coordinates and isotropic or equivalent isotropic displacement parameters (Å^2^)

**Table:**

	*x*	*y*	*z*	*U*_iso_*/*U*_eq_	
Cl1	0.31149 (3)	1.03231 (4)	0.23458 (3)	0.02272 (14)	
O1	0.37595 (8)	0.96775 (12)	0.89939 (8)	0.0230 (3)	
N1	0.30801 (9)	0.93219 (13)	0.64941 (10)	0.0165 (3)	
C1	0.41986 (11)	0.89933 (18)	0.72853 (12)	0.0199 (3)	
H1A	0.4258	0.7859	0.7509	0.024*	
H1B	0.4718	0.9204	0.6870	0.024*	
C2	0.44732 (12)	1.00099 (18)	0.83543 (12)	0.0220 (3)	
H2A	0.5218	0.9794	0.8838	0.026*	
H2B	0.4420	1.1146	0.8135	0.026*	
C3	0.27034 (12)	1.00809 (18)	0.83116 (13)	0.0227 (3)	
H3A	0.2674	1.1225	0.8116	0.027*	
H3B	0.2208	0.9892	0.8760	0.027*	
C4	0.23423 (11)	0.91112 (18)	0.72105 (12)	0.0201 (3)	
H4A	0.2319	0.7974	0.7407	0.024*	
H4B	0.1610	0.9438	0.6752	0.024*	
C5	0.28285 (11)	0.80630 (17)	0.55463 (12)	0.0198 (3)	
H5A	0.3283	0.8261	0.5054	0.024*	
H5B	0.3025	0.7008	0.5908	0.024*	
C6	0.16760 (11)	0.80131 (16)	0.47930 (12)	0.0182 (3)	
C7	0.09501 (12)	0.70572 (17)	0.51050 (13)	0.0224 (3)	
H7	0.1174	0.6504	0.5817	0.027*	
C8	−0.00926 (12)	0.69048 (18)	0.43888 (13)	0.0260 (4)	
H8	−0.0586	0.6273	0.4620	0.031*	
C9	−0.04183 (12)	0.76705 (18)	0.33380 (13)	0.0263 (4)	
H9	−0.1128	0.7536	0.2834	0.032*	
C10	0.02947 (12)	0.86372 (19)	0.30209 (13)	0.0268 (4)	
H10	0.0068	0.9180	0.2304	0.032*	
C11	0.13372 (12)	0.88136 (18)	0.37469 (12)	0.0224 (3)	
H11	0.1820	0.9483	0.3529	0.027*	
C12	0.29561 (12)	1.09797 (17)	0.59839 (12)	0.0227 (3)	
H12A	0.2232	1.1076	0.5417	0.027*	
H12B	0.3008	1.1757	0.6606	0.027*	
C13	0.37708 (14)	1.1417 (2)	0.53976 (13)	0.0328 (4)	
H13A	0.4493	1.1329	0.5950	0.049*	
H13B	0.3646	1.2512	0.5113	0.049*	
H13C	0.3700	1.0695	0.4751	0.049*	

### Atomic displacement parameters (Å^2^)

**Table:**

	*U*^11^	*U*^22^	*U*^33^	*U*^12^	*U*^13^	*U*^23^
Cl1	0.0235 (2)	0.0170 (2)	0.0296 (2)	−0.00200 (13)	0.01123 (17)	−0.00037 (14)
O1	0.0241 (6)	0.0242 (6)	0.0192 (5)	−0.0001 (4)	0.0046 (5)	0.0020 (4)
N1	0.0168 (6)	0.0128 (6)	0.0188 (6)	−0.0008 (5)	0.0043 (5)	−0.0006 (5)
C1	0.0151 (7)	0.0203 (8)	0.0219 (7)	0.0008 (6)	0.0023 (6)	0.0000 (6)
C2	0.0194 (7)	0.0230 (8)	0.0213 (7)	−0.0024 (6)	0.0035 (6)	−0.0002 (6)
C3	0.0225 (8)	0.0234 (8)	0.0227 (8)	−0.0002 (6)	0.0080 (6)	−0.0017 (6)
C4	0.0178 (7)	0.0189 (8)	0.0250 (8)	−0.0014 (6)	0.0086 (6)	−0.0012 (6)
C5	0.0205 (7)	0.0140 (7)	0.0243 (7)	−0.0012 (6)	0.0065 (6)	−0.0044 (6)
C6	0.0195 (7)	0.0127 (7)	0.0216 (7)	0.0000 (6)	0.0054 (6)	−0.0052 (6)
C7	0.0266 (8)	0.0139 (7)	0.0247 (8)	−0.0027 (6)	0.0053 (7)	0.0001 (6)
C8	0.0223 (8)	0.0187 (8)	0.0362 (9)	−0.0044 (6)	0.0082 (7)	−0.0017 (7)
C9	0.0203 (7)	0.0208 (8)	0.0325 (8)	0.0022 (6)	0.0007 (7)	−0.0042 (7)
C10	0.0298 (8)	0.0251 (9)	0.0221 (8)	0.0047 (7)	0.0034 (7)	0.0010 (6)
C11	0.0262 (8)	0.0200 (8)	0.0226 (8)	−0.0018 (6)	0.0100 (7)	−0.0017 (6)
C12	0.0305 (8)	0.0122 (7)	0.0210 (7)	−0.0014 (6)	0.0020 (6)	0.0012 (6)
C13	0.0501 (10)	0.0252 (9)	0.0227 (8)	−0.0146 (8)	0.0108 (8)	0.0002 (7)

### Geometric parameters (Å, °)

**Table:**

O1—C3	1.4191 (18)	C5—H5B	0.9900
O1—C2	1.4300 (17)	C6—C11	1.391 (2)
N1—C1	1.5111 (18)	C6—C7	1.393 (2)
N1—C4	1.5129 (18)	C7—C8	1.382 (2)
N1—C12	1.5167 (18)	C7—H7	0.9500
N1—C5	1.5290 (18)	C8—C9	1.381 (2)
C1—C2	1.510 (2)	C8—H8	0.9500
C1—H1A	0.9900	C9—C10	1.388 (2)
C1—H1B	0.9900	C9—H9	0.9500
C2—H2A	0.9900	C10—C11	1.388 (2)
C2—H2B	0.9900	C10—H10	0.9500
C3—C4	1.519 (2)	C11—H11	0.9500
C3—H3A	0.9900	C12—C13	1.515 (2)
C3—H3B	0.9900	C12—H12A	0.9900
C4—H4A	0.9900	C12—H12B	0.9900
C4—H4B	0.9900	C13—H13A	0.9800
C5—C6	1.507 (2)	C13—H13B	0.9800
C5—H5A	0.9900	C13—H13C	0.9800
			
C3—O1—C2	108.83 (11)	C6—C5—H5B	108.6
C1—N1—C4	106.37 (10)	N1—C5—H5B	108.6
C1—N1—C12	112.87 (11)	H5A—C5—H5B	107.6
C4—N1—C12	110.00 (10)	C11—C6—C7	119.02 (14)
C1—N1—C5	107.05 (11)	C11—C6—C5	121.18 (13)
C4—N1—C5	109.58 (10)	C7—C6—C5	119.63 (13)
C12—N1—C5	110.82 (11)	C8—C7—C6	120.69 (14)
C2—C1—N1	111.70 (12)	C8—C7—H7	119.7
C2—C1—H1A	109.3	C6—C7—H7	119.7
N1—C1—H1A	109.3	C9—C8—C7	120.09 (14)
C2—C1—H1B	109.3	C9—C8—H8	120.0
N1—C1—H1B	109.3	C7—C8—H8	120.0
H1A—C1—H1B	107.9	C8—C9—C10	119.78 (14)
O1—C2—C1	110.26 (12)	C8—C9—H9	120.1
O1—C2—H2A	109.6	C10—C9—H9	120.1
C1—C2—H2A	109.6	C11—C10—C9	120.23 (14)
O1—C2—H2B	109.6	C11—C10—H10	119.9
C1—C2—H2B	109.6	C9—C10—H10	119.9
H2A—C2—H2B	108.1	C10—C11—C6	120.17 (14)
O1—C3—C4	111.57 (12)	C10—C11—H11	119.9
O1—C3—H3A	109.3	C6—C11—H11	119.9
C4—C3—H3A	109.3	C13—C12—N1	114.80 (13)
O1—C3—H3B	109.3	C13—C12—H12A	108.6
C4—C3—H3B	109.3	N1—C12—H12A	108.6
H3A—C3—H3B	108.0	C13—C12—H12B	108.6
N1—C4—C3	112.00 (11)	N1—C12—H12B	108.6
N1—C4—H4A	109.2	H12A—C12—H12B	107.5
C3—C4—H4A	109.2	C12—C13—H13A	109.5
N1—C4—H4B	109.2	C12—C13—H13B	109.5
C3—C4—H4B	109.2	H13A—C13—H13B	109.5
H4A—C4—H4B	107.9	C12—C13—H13C	109.5
C6—C5—N1	114.73 (11)	H13A—C13—H13C	109.5
C6—C5—H5A	108.6	H13B—C13—H13C	109.5
N1—C5—H5A	108.6		
			
C4—N1—C1—C2	−54.32 (14)	N1—C5—C6—C11	95.68 (15)
C12—N1—C1—C2	66.40 (15)	N1—C5—C6—C7	−89.09 (16)
C5—N1—C1—C2	−171.41 (11)	C11—C6—C7—C8	−0.1 (2)
C3—O1—C2—C1	−62.64 (15)	C5—C6—C7—C8	−175.43 (13)
N1—C1—C2—O1	61.35 (15)	C6—C7—C8—C9	1.7 (2)
C2—O1—C3—C4	61.11 (15)	C7—C8—C9—C10	−2.2 (2)
C1—N1—C4—C3	52.26 (15)	C8—C9—C10—C11	1.1 (2)
C12—N1—C4—C3	−70.29 (15)	C9—C10—C11—C6	0.5 (2)
C5—N1—C4—C3	167.65 (12)	C7—C6—C11—C10	−1.0 (2)
O1—C3—C4—N1	−57.89 (16)	C5—C6—C11—C10	174.27 (13)
C1—N1—C5—C6	169.51 (11)	C1—N1—C12—C13	52.59 (16)
C4—N1—C5—C6	54.55 (15)	C4—N1—C12—C13	171.21 (12)
C12—N1—C5—C6	−67.02 (15)	C5—N1—C12—C13	−67.46 (15)

### Hydrogen-bond geometry (Å, °)

**Table:**

*D*—H···*A*	*D*—H	H···*A*	*D*···*A*	*D*—H···*A*
C1—H1B···Cl1^i^	0.99	2.74	3.4724 (16)	131
C5—H5B···Cl1^ii^	0.99	2.61	3.5500 (16)	158
C11—H11···Cl1	0.95	2.66	3.5501 (16)	156
C12—H12B···Cl1^iii^	0.99	2.61	3.5062 (16)	151

Symmetry codes: (i) −*x*+1, −*y*+2, −*z*+1; (ii) *x*, −*y*+3/2, *z*+1/2; (iii) *x*, −*y*+5/2, *z*+1/2.
